# Obesity and Early‐Stage Breast Cancer: A Comprehensive Analysis of Tumor Biology, Staging Accuracy, and Long‐Term Outcomes

**DOI:** 10.1002/osp4.70098

**Published:** 2025-11-18

**Authors:** Marco Yusef, Augusto Lombardi, Valeria Vitale, Gianluca Stanzani, Niccolò Petrucciani, Francesco Maria Carrano, Francesco Spinelli, Danila Capoccia, Gianfranco Silecchia

**Affiliations:** ^1^ Department of Medical and Surgical Sciences and Translational Medicine Faculty of Medicine and Psychology St Andrea University Hospital Sapienza University Rome Italy; ^2^ General Surgery Residency Program Faculty of Medicine and Psychology Sapienza University of Rome Rome Italy; ^3^ Breast Unit S. Andrea University Hospital Rome Italy; ^4^ Department of Medico‐surgical Sciences and Biotechnologies Faculty of Pharmacy and Medicine Santa Maria Goretti Hospital of Latina Sapienza University Rome Italy

**Keywords:** axillary staging, breast cancer, early stagen, obesity, premenopausal, weight stigma

## Abstract

**Background:**

Obesity increases the risk of breast cancer by approximately 10% for every 5‐unit increase in body mass index (BMI), underscoring its role as a key modifiable risk factor.

**Objective:**

To determine the prevalence of obesity among early‐stage breast cancer patients at an academic breast unit and to evaluate its association with tumor characteristics, surgical outcomes, and survival, with a particular focus on premenopausal women.

**Methods:**

Prospectively collected data from patients treated between January 2005 and December 2023 were retrospectively analyzed. Statistical analyses, including chi‐squared tests, ANOVA, Kaplan–Meier, and univariate regression analyses, were used to assess obesity prevalence, tumor size, axillary staging accuracy, molecular subtypes, surgical margins, recurrence, survival, and menopausal status.

**Results:**

Among 1187 patients, 55.6% were normal weight, 25.4% were overweight, and 19% were living with obesity (BMI > 30). Obesity was more common in patients > 50 years of age (22% vs. 10%; *p* = 0.001). Mean tumor size increased with BMI (13.8 vs. 15.4 mm vs. 17.6 mm; *p* = 0.001). Notably, in premenopausal patients, obesity correlated with a higher proportion of poorly differentiated (G3) tumors (50% vs. 27%; *p* < 0.05), while survival remained similar across BMI categories.

**Conclusion:**

Obesity is associated with larger tumors, poorer axillary staging, and a higher prevalence of aggressive histological subtypes among premenopausal patients. However, obesity did not significantly influence overall survival. Targeted screening and multidisciplinary management strategies are essential to improve early detection and individualize treatment for patients with obesity.

## 1 Introduction

Breast cancer remains the leading cause of cancer‐related mortality among women worldwide, with approximately 2.3 million new diagnoses and 685,000 deaths reported in 2020 [1, 2, 3, 4]. In Italy alone, an estimated 55,000 new cases are reported annually [5]. Established risk factors include advanced age, family history, and hereditary genetic mutations [5]. However, obesity has emerged as a major modifiable risk factor influencing both the incidence and progression of breast cancer.

Epidemiological evidence suggests that each 5‐unit increase in body mass index (BMI) is associated with an approximately 10% increase in breast cancer risk [1, 6, 7], although this relationship is strongly influenced by menopausal status. For instance, a meta‐analysis of 34 datasets encompassing over 2.5 million women reported an 8% reduction in breast cancer risk per 5 kg/m^2^ increase in BMI among premenopausal women, whereas risk increased in postmenopausal population [8]. Similarly, the Million women Study, which followed 1.2 million women aged 50–64 for an average of 5.4 years and identified 45,037 breast cancer cases, demonstrated that postmenopausal women with obesity had approximately a 30% higher risk of developing breast cancer compared with their counterparts without obesity [9].

Women with obesity also face unique challenges in breast cancer screening, partly due to weight‐related stigma and technical limitations in imaging techniques [10, 11, 12, 13, 14, 15, 16, 17]. These barriers may contribute to delayed diagnosis and more advanced disease at presentation [18, 19]. Although accumulating evidence links obesity with adverse breast cancer outcomes [20], data from Italy remain limited, particularly regarding differences between premenopausal and postmenopausal populations.

The interplay between obesity and cancer has garnered increasing attention across multiple oncologic domains. Beyond breast cancer, emerging data underscore the role of excess adiposity in modulating both the risk and progression of various malignancies, including gynecologic, pancreatic, and obesity‐related cancers in older women. For example, Pavone et al. (2024) [21] explored the so‐called “obesity paradox” in gynecologic cancers, suggesting a potential survival advantage among patients with higher BMI. In preclinical studies, Wirkus et al. (2025) [22] demonstrated that dietary normalization of body weight can mitigate obesity‐driven pancreatic carcinogenesis. Furthermore, Abdelgadir et al. (2025) [23] highlighted the potential benefits of pharmacologic weight management, reporting a reduced cancer risk among older women with obesity treated with anti‐obesity medications. Collectively, these findings situate the present study within a broader oncologic framework, underscoring the importance of addressing obesity not only as a metabolic disorder but also as a modifiable determinant of cancer risk and outcome across tumor types.

The aim of this study was to evaluate the prevalence of obesity in patients with early‐stage (N0) breast cancer treated at a tertiary academic breast center. Specifically, we examined associations between BMI categories and tumor characteristics‐including size, histologic grade, and molecular profile‐as well as surgical outcomes and survival. The underlying hypothesis is that obesity correlates with larger tumor dimensions and more aggressive histopathologic features, particularly in premenopausal women. Ultimately, this research seeks to identify potential preventive strategies that may facilitate earlier detection and improve clinical outcomes in obese women with breast cancer. In addition, the findings may serve as a foundation for future prospective studies aimed at furthering the understanding of the impact of obesity on breast cancer.

## 2 Materials and Methods

A retrospective, single‐center, observational study was conducted using prospectively collected data from patients treated at a tertiary referral center for breast surgery between January 2005 and December 2023. All participants provided written informed consent permitting the use of their clinical data for research purposes.

Inclusion criteria:

— Female patients aged > 18 years.
— First diagnosis of breast cancer (any receptor status, molecular profile, or histotype).
— Staged as N0 at preoperative evaluation (axillary ultrasound with or without cytological confirmation).
— Provision of written informed consent for research purposes.

Exclusion criteria:

— Preoperative evidence of lymph node positivity.
— Participation in clinical trials involving experimental drugs within 1 year prior to recruitment or concurrently in trials that might confound results.
— Incomplete electronic records.
— Patients with known BRCA, PIK3CA, TP53 mutations.

## 3 Data Collection

Relevant clinical, pathological, and surgical data were extracted anonymously from electronic medical records and transferred into a secure REDCap database. Patients were categorized according to BMI as follows:

1. Normal weight (BMI 18.5–24.9 kg/m^2^).
2. Overweight (BMI 25–30 kg/m^2^).
3. Obese (BMI > 30 kg/m^2^)

### 3.1 Endpoints

- Primary Endpoint: Prevalence of obesity in early‐stage breast cancer patients.
- Secondary Endpoints:
  ◦ Comparison of tumor size, axillary staging accuracy (false negative rate), molecular subtypes, and tumor stage by BMI.
  ◦ Analysis of surgical outcomes (type of surgery, margin status).
  ◦ Assessment of recurrence and survival outcomes.
  ◦ Evaluation of the impact of pre‐ and postmenopausal status on these parameters.

### 3.2 Statistical Analysis

Categorical variables were compared using the chi‐squared test, and continuous variables were analyzed using ANOVA. Kaplan–Meier survival analysis was performed for overall and breast cancer‐specific survival. Statistical significance was set at *p* < 0.05 (with *p* < 0.01 considered highly significant). Analyses were performed with SPSS version 25.

## 4 Results

A total of 1187 patients fulfilled the inclusion criteria. The overall mean age was 60 years (range 30–93, ± 12.79) (Table 1). The BMI distribution was as follows: 55.6% of patients had a BMI < 25, 25.4% had a BMI between 25% and 30%, and 19% had a BMI > 30. Analysis of histologic tumor diameter stratified by BMI class (Table 1) shows that normal weight patients had a mean tumor size of 13.8 (± 8.3) mm, while overweight patients had a mean tumor size of 15.4 (± 9.4) mm and patients with obesity had a mean tumor size of 17.6 (± 10.9) mm (*p* = 0.001). Obesity was significantly more prevalent in patients over 50 years of age (22%) compared to those under 50 years of age (10%; *p* = 0.001), as detailed in Table 1. All patients were initially staged as N0. The false negative rate for preoperative axillary staging was 22.9% in patients with BMI < 25, 22.6% in patients with BMI 25%‐30%, and 30% in obese patients (*p* = NS). No statistically significant differences were observed in the distribution of molecular subtypes (luminal A, luminal B, HER2+, triple negative) in the obese population compared to the normal weight population. Although normal weight patients had a higher rate of mastectomy (8.3%), obese patients were more likely to undergo quadrantectomy (*p* < 0.034) (Table 1).

**TABLE 1:** Patient demographics, surgical interventions, and tumor features stratified by BMI.

Variable	BMI < 25	BMI 25–30	BMI > 30	*p*
Patient weight	55.6%	25.36%	55.6%	
Tumor size (14.91 ± 9274)	13.76 ± 8.3	15.41 ± 9.4	17.61 ± 10.9	0.001
Patient > 50 years (872 pz 73%)	48.90%	29.00%	22.10%	0.001
Patient < 50 years (315 pz 27%)	74.30%	15.20%	10.50%	0.001
Preoperative axillary staging false negatives (287, 24.2%)	22.90%	22.60%	30.10%	0.26
Tumor luminal A–B (736; 261)	412; 141	182; 69	142; 51	Not significant
Tumor luminal her2‐TN (121; 63)	68; 34	31; 19	22; 10	Not significant
Type of surgery (mastectomy 153–12.9%)	8.3%	2.9%	1.7%	
Type of surgery (quad. 153–86.9%)	47%	22%	17.3%	
R1 (97%–8.2%)	63.80%	23.40%	12.80%	0.33

A non‐significant trend toward higher rates of R1 resections was observed in normal weight patients compared to obese patients. The rate of R1 was 5.8% in patients with a BMI less than 25, 2.1% in patients with a BMI between 25% and 30%, and 1.2% in patients with a BMI greater than 30 (Table 1).

**FIGURE 1:**
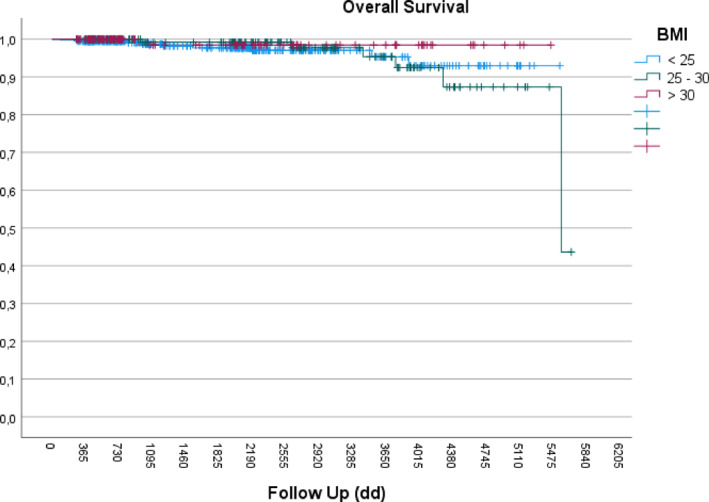
Kaplan–Meier curves showing > 95% overall survival at 10 years across all BMI groups.

**FIGURE 2:**
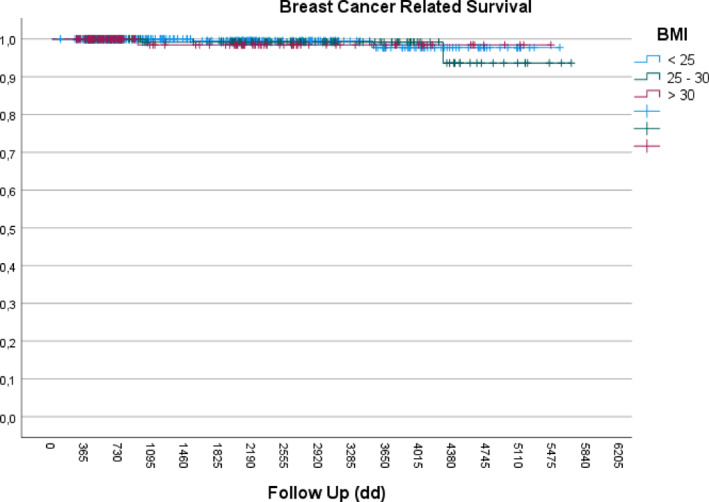
Kaplan–Meier curves showing breast cancer‐specific survival of nearly 100% across all BMI groups.

At 10 years, overall survival was > 95% and breast cancer‐specific survival was nearly 100% in all BMI groups (Figures 1 and 2), with no statistically significant differences observed. In patients with luminal histology, there were no significant differences in tumor size (T), axillary staging (N), survival and recurrence curves stratified by BMI compared to analyses already performed considering all tumor types (her2+ and triple negative).

Among premenopausal patients, obese women had a significantly higher proportion of poorly differentiated (G3) tumors (50%) compared to those with a lower BMI (approximately 26%–27%; *p* < 0.05) (Table 2).

**TABLE 2:** Tumor grading variations according to BMI.

			Grading		Tot
			G1–G2	G3	
BMI	< 25	Number	171	62	233
		% In BMI	73.4%	26.6%	100.0%
		% In G_class	77.4%	68.1%	74.7%
		% Tot	54.8%	19.9%	74.7%
	25–30	Number	34	13	47
		% In BMI	72.3%	27.7%	100.0%
		% In G_class	15.4%	14.3%	15.1%
		% Tot	10.9%	4.2%	15.1%
	> 30	Number	16	16	32
		% In BMI	50.0%	50.0%	100.0%
		% In G_class	7.2%	17.6%	10.3%
		% Tot	5.1%	5.1%	10.3%

## 5 Discussion

The present study investigated the prevalence and clinical impact of obesity in patients with early (N0) breast cancer treated at a tertiary academic center over an 18‐year period. The data showed that 19% of the cohort were classified as living with obesity, a proportion almost double that observed in the general Italian female population. The stigma associated with obesity, as documented in the literature, may contribute to lower adherence to routine breast cancer screening programs, thereby delaying diagnosis and initial clinical management.

Histopathological analysis revealed that patients with obesity presented with significantly larger primary tumors compared to their normal‐weight and overweight counterparts. This disparity may lead to more advanced pathological staging at diagnosis and, consequently, less favorable prognostic outcomes. These differences are likely driven by a combination of delayed detection and the biological effects of excess adiposity on tumor growth and progression, further compounded by imaging challenges associated with increased breast volume.

Age stratification using a 50‐year threshold showed that 73% of patients were postmenopausal, while 27% were premenopausal. The prevalence of obesity was significantly higher in the older subgroup, with 22% classified as obese compared to 10% of younger women.

Preoperative axillary staging, performed according to established clinical guidelines ‐ using ultrasound and lymph node cytology when indicated [5] ‐ showed a trend toward higher false negative rates in patients with obesity (30.0%) compared to patients without obesity (22.9%). This discrepancy is likely attributable to the technical limitations of axillary imaging in individuals with higher body mass.

Analysis of tumor receptor status ‐ an important determinant of treatment strategy ‐ showed no statistically significant differences in the distribution of molecular subtypes (luminal A, luminal B, HER2‐positive, triple‐negative) across BMI categories. These findings suggest that although obesity appears to affect tumor size and stage at presentation, it may not substantially alter the intrinsic biological characteristics of the tumor.

Paradoxically, despite presenting with larger tumors, patients with obesity were more likely to be treated with breast‐conserving surgery, such as quadrantectomy, rather than mastectomy. This observation may reflect the technical feasibility of breast‐conserving surgery in patients with obesity, given the greater volume of residual breast tissue. However, the lack of data on reconstruction after mastectomy limits a comprehensive assessment of surgical outcomes in relation to BMI.

Margin status analysis showed a non‐significant trend toward a higher incidence of R1 resections in normal weight patients, possibly due to a less favorable tumor‐to‐breast tissue ratio. In terms of tumor differentiation, although no overall differences were observed in the overall cohort, subgroup analysis identified a significantly higher prevalence of poorly differentiated (G3) tumors in obese premenopausal women. While the World Health Organization (WHO) has reported a strong association between obesity and adverse histological features in postmenopausal breast cancer [24], evidence in premenopausal populations remains inconclusive. It has been hypothesized that postmenopausal increases in visceral adiposity may elevate circulating estrogen levels, thereby promoting breast cancer risk, whereas the premenopausal hormonal milieu—characterized by anovulatory cycles and lower progesterone levels—may provide some protective effect [25]. Nevertheless, the findings of the present study suggest that obesity may be linked to a more aggressive tumor phenotype even among premenopausal patients.

Ten‐year survival analysis stratified by BMI category showed no statistically significant differences between normal weight, overweight, and patients with obesity. This observation may reflect a compensatory interplay between adverse prognostic factors, such as larger tumor size and more advanced stage, and favorable surgical outcomes associated with breast‐conserving procedures. It may also be attributable to the study's exclusive focus on early‐stage (N0) disease, which inherently carries a more favorable prognosis, as documented by WHO data [24].

Several limitations must be acknowledged. The retrospective and single‐institution design may limit the generalizability of the findings. In addition, the extended study period (2005–2023) includes significant advances in diagnostic and therapeutic protocols that may have influenced the results. While the focus on early‐stage disease allowed for a more homogeneous cohort, it limits the applicability of the findings to more advanced breast cancer cases.

Future research should prioritize prospective, multicenter studies to validate these findings and to further elucidate the pathophysiological mechanisms linking obesity to breast cancer aggressiveness. In parallel, personalized screening protocols and weight‐loss interventions should be systematically evaluated for their impact on tumor characteristics and clinical outcomes. Addressing the technical limitations of imaging in patients with obesity may also improve early detection and diagnostic accuracy. Importantly, research should extend beyond preventive strategies for at‐risk populations to include obese individuals already diagnosed with breast cancer. For these patients, the identification of effective weight loss interventions ‐ including dietary modification and bariatric surgery ‐ and the optimal timing of such interventions remain essential.

Breast cancer remains a major public health challenge for women, often affecting younger populations and contributing significantly to morbidity. The increasing prevalence of obesity, a well‐established risk factor, may further increase this burden. The results of the present study highlight the need for tailored screening strategies for women with obesity to facilitate earlier diagnosis and improved clinical outcomes. In addition, interdisciplinary collaboration between breast oncology and obesity specialists is essential to provide comprehensive, individualized care.

## Funding

The authors have nothing to report.

## Conflicts of Interest

The authors declare no conflicts of interest.

## Data Availability

The data that support the findings of this study are available on request from the corresponding author. The data are not publicly available due to privacy or ethical restrictions.
